# Motor Fault Diagnosis Under Strong Background Noise Based on Parameter-Optimized Feature Mode Decomposition and Spatial–Temporal Features Fusion

**DOI:** 10.3390/s25134168

**Published:** 2025-07-04

**Authors:** Jingcan Wang, Yiping Yuan, Fangqi Shen, Caifeng Chen

**Affiliations:** 1 School of Mechanical Engineering, Xinjiang University, Urumqi 830017, China; 107552304442@stu.xju.edu.cn (J.W.); ccf@stu.xju.edu.cn (C.C.); 2 School of Business, University of Leeds, Leeds LS2 9JT, UK; lskj0227@leeds.ac.uk

**Keywords:** parameter-optimized feature mode decomposition, strong background noise, dual-channel model, motor fault diagnosis

## Abstract

As the mining motor is used long-term in a complex multi-source noise environment composed of equipment group coordinated operations and high-frequency start–stop, its vibration signal has the features of significant strong noise interference, weak fault features, and the superposition of multiple working conditions coupling, which makes it arduous to efficiently extract and identify mechanical fault features. To address this issue, this study introduces a high-performance fault diagnosis approach for mining motors operating under strong background noise by integrating parameter-optimized feature mode decomposition (WOA-FMD) with the RepLKNet-BiGRU-Attention dual-channel model. According to the experimental results, the average accuracies of the proposed method were 97.7% and 93.38% for the noise-added CWRU bearing fault dataset and the actual operation dataset of the mine motor, respectively, which are significantly better than those of similar methods, showing that the approach in this study is superior in fault feature extraction and identification.

## 1. Introduction

Because of the severe and intricate working conditions in mines, motors continuously operate under dust, moisture, and heavy load conditions. Electrical and mechanical failures are prone to occur, and mechanical failures can have an irreversible impact on the structural reliability of the motor; thus, achieving efficient and precise mechanical fault diagnosis for motors is crucial for maintaining overall equipment safety and stability.

Motor fault diagnosis primarily comprises two key processes: extracting fault features and recognizing fault patterns. Owing to the special working conditions, such as the cooperative operation of equipment groups and high-frequency starting and stopping of the power system at the mining site, the vibration signal of the motor is often characterized by strong noise inundation, weak fault features, and the coupling of working conditions. This strong noise interference seriously drowns out weak early mechanical fault features. To ensure accurate fault diagnosis results, the vibration signal must undergo a noise-reduction process. Various approaches have been used to eliminate background noise interference, with signal decomposition methods among the most widely used, such as empirical mode decomposition (EMD) [1,2], ensemble empirical mode decomposition (EEMD) [3,4], local mean decomposition (LMD) [5,6], local characteristic-scale decomposition (LCD) [7], symplectic geometry mode decomposition (SGMD) [8], variational mode decomposition (VMD) [9,10,11], etc. However, all of these methods are susceptible to problems such as mode aliasing and boundary effects. To address the aforementioned problems and overcome the limitations of current signal decomposition algorithms, as well as to enhance fault diagnosis, Miao et al. [12] introduced feature mode decomposition (FMD), which employs an adaptive finite impulse response (FIR) filter to iteratively break down signals and efficiently uncover hidden fault features. However, FMD lacks parameter adaptability and requires the manual setting of three crucial parameters: the count of modal decompositions (*n*), filter length (L), and the number of band divisions (K). Unreasonable parameter settings can seriously affect the decomposition performance [13]. Therefore, optimizing the three parameters of FMD is essential for improving its ability to extract fault features, particularly in the presence of strong background noise. In current research on FMD parameter optimization, most studies have employed optimization algorithms to identify optimal parameter combinations, leading to improved decomposition results [14,15,16].

Efficient classification models are required for fault identification, along with the extraction of fault signal features. Intelligent fault diagnosis methods based on deep learning are extensively used in machinery fault diagnosis because they can automatically learn data features without requiring specialized expertise [17]. Classical deep learning models include CNN, LSTM, and GRU. The 1D-CNN captures the temporal and spatial features of the signal by directly performing a 1D convolution operation on the time series. However, it fails to obtain spatial relationships within the series. The 2D-CNN effectively captures local relationships within images and demonstrates superior capability in characterizing and learning spatial features. LSTM and GRU capture long-term dependencies in the signals [18]. Wang et al. [19] utilized a short-time Fourier transform (STFT) to generate time–frequency images and then employed a 2D-CNN to extract fault features for motor fault diagnosis. Gu et al. [20] used the VMD decomposition method to decompose the signal and reconstruction, and then time–frequency images generated through continuous wavelet transform (CWT) were fed into a 2D-CNN model to achieve fault classification. Wang et al. [21] synthesized the advantages of the above classical models and proposed a CNN-GRU network. The CNN captures local features and the GRU captures the global features of the time series to achieve fault classification. Chen et al. [22] utilized a deep graph convolutional network (DGCN) to classify rolling-bearing composite faults and achieved ideal results. Lv et al. [23] constructed a high-precision fault diagnosis architecture by combining FMD-processed signals with the transformer’s outstanding ability to learn global features. Qin et al. [24,25,26] designed several anti-noise feature fusion fault diagnosis networks using different methods for the problem of noise interference and sample scarcity in complex industrial scenarios and achieved good classification results. Although the aforementioned fault diagnosis models have achieved excellent performance, they all rely solely on a single 1D or 2D model and data type, making it difficult to extract features simultaneously from both 1D time-series data and 2D image data effectiveness in feature extraction.

Based on the research methods mentioned above, this study combines WOA-FMD with the RepLKNet-BiGRU-Attention dual-channel model to propose a new fault diagnosis method for mining motors under strong background noise. First, the WOA-FMD algorithm was used to adaptively decompose the raw signal, eliminating the non-periodic noise components contained in the signal. Then, the kurtosis of the squared envelope spectrum (KSES) index was applied to select intrinsic mode functions (IMFs) with extensive fault-related features for signal reconstruction. Finally, the signal is transformed into a time–frequency image using CWT and fed into the dual-channel RepLKNet-BiGRU-Attention fault diagnosis model along with the one-dimensional signal to extract the temporal and spatial features of the signal, thereby achieving fault diagnosis for mining motors.

The main contributions of this study are as follows:

1. Data decomposition stage: Aiming at the lack of parameter adaptability of FMD, which leads to insufficient signal decomposition performance, the WOA optimization algorithm is introduced to perform the adaptive optimization of three key parameters [*n*, L, K], which solves the problem of the error caused by relying on the subjective experience of parameter selection and effectively removes the stochastic noise components in the signal.
2. Signal reconstruction stage: To address the issue of the inaccurate selection of dominant IMFs, the average KSES value of all IMFs was used as a threshold to filter out IMFs with prominent fault features. The raw vibration signal was then reconstructed, effectively removing irrelevant noise components such as white noise and mechanical vibration noise, thereby minimizing their impact on the subsequent model diagnosis results.
3. Fault Mode Recognition: To overcome the drawback of single-data-type models in comprehensively capturing signal characteristics, this study introduces the RepLKNet-BiGRU-Attention dual-channel architecture. Continuous wavelet transform (CWT) is employed to create time–frequency images, which are subsequently input into the RepLKNet component for extracting intricate spatial features within the signal. Concurrently, the one-dimensional signal is fed into the BiGRU-Attention component to capture its temporal dependencies. This mechanism of multi-feature fusion allows for the concurrent extraction of both temporal dynamics and spatial patterns from input signals, thereby notably enhancing the representation of fault features and boosting the overall performance of the model.
4. Experimental Validation: To evaluate the performance of the method, experiments were carried out on a noise-added CWRU bearing fault dataset and actual operating data from mining motors. The results show that the method substantially improves motor fault diagnosis performance under strong background noise.

The subsequent part of this study is structured as follows: Section 2 details the proposed theoretical framework and the overall architecture of the fault diagnosis method. Section 3 demonstrates the effectiveness and advantages of the proposed approach through two case studies. Finally, Section 4 summarizes the conclusions of this study and outlines future research directions.

## 2. Theoretical Methodology

This section elaborates on the key theoretical methods used for feature extraction and pattern recognition. These include signal decomposition and reconstruction based on WOA-FMD, 2D time–frequency image generation based on CWT, and a dual-channel fault recognition model based on RepLKNet-BiGRU-Attention.

### 2.1. Signal Decomposition and Reconstruction Based on WOA-FMD

#### 2.1.1. FMD

FMD obtains multiple modes by designing an adaptive finite impulse response (FIR). FMD is a non-recursive approach that utilizes correlation kurtosis as its objective function and filters noisy signals by building an FIR and updating the filter coefficients to achieve the extraction of impact signals. The implementation of the FMD method consists of three core aspects: the initialization of the adaptive FIR filter bank, the adaptive updating of the filter coefficients, and the screening of the modal components. First, the original signal spectrum is divided into *K* subbands at equal intervals and the entire target frequency band is completely covered using a uniformly distributed FIR filter bank, which is mathematically represented as:

(1) $$\left\{\begin{array}{c} f_{k}={\left[f_{k}(1)\cdots f_{k}(l)\cdots f_{k}(L)\right]}^{T}\\ f_{l}=k\cdot f_{s}/2K\\ f_{u}=(k+1)\cdot k\cdot f_{s}/2K \end{array}\right.$$

where *f_s_* is the signal sampling frequency; *f_k_* denotes the kth filter of length *L*; and *f_l_* and *f_u_* correspond to the lower and upper cutoff frequencies of the filter, respectively. In order to suppress the dispersion effect of fault components among modes, FMD models the decomposition process as a constrained optimization problem with the following mathematical expression:

(2) $$\left\{\begin{array}{c} \underset{\left\{f_{k}(l)\right\}}{\arg\max}\left\{\begin{array}{l} CK_{M}\left(u_{k}\right)=\sum_{n=1}^{N}{\left(\prod_{m=0}^{M}u_{k}\left(n-mT_{s}\right)\right)}^{2}/\\ {\left(\sum_{n=1}^{N}u_{k}{\left(n\right)}^{2}\right)}^{M+1} \end{array}\right\}\\ \;s.t.\;u_{k}(n)=\sum_{l=1}^{L}f_{k}(l)x(n-l+1) \end{array}\right.$$

where *u_k_*(*n*) denotes the *k*th modal component; *T_s_* is the period of the input signal measured by the number of samples; and *M* is the number of shifts. The constrained optimization problem is solved by the iterative eigenvalue decomposition algorithm with the following expression for the modal components:

(3) $$U_{k}=Xf_{k}$$

(4) $$U_{k}=\left[\begin{array}{c} u_{k}(1)\\ \vdots \\ u_{k}(N-L+1) \end{array}\right]$$

(5) $$X=\left[\begin{array}{ccc} x(1) & \ldots & x(L)\\ \vdots & \ddots & \vdots \\ x(N-L+1) & \ldots & x(N) \end{array}\right]$$

**Figure 1:**
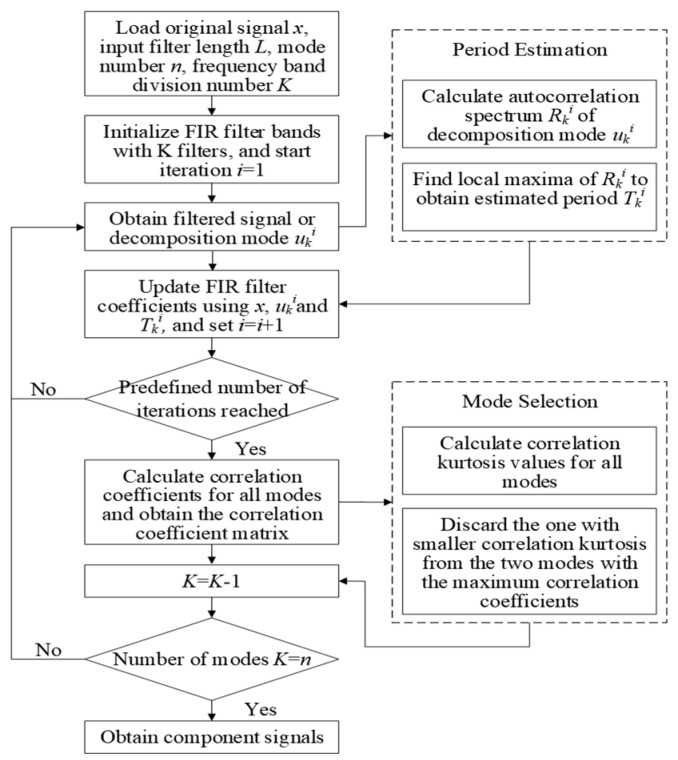
The implementation process of the FMD method.

FMD adopts the correlated kurtosis (CK) maximization criterion to filter modal components and locks the dominant modes based on the maximum correlation coefficient (C_C_), while excluding components with low CK values to enhance the fault feature retention effect. The correlation coefficient matrix is defined as:

(6) $$C_{C_{\mathrm{pq}}}=\frac{\sum_{n=1}^{N}\left(u_{p}(n)-{\bar{u}}_{p}\right)\left(u_{q}(n)-{\bar{u}}_{q}\right)}{\sqrt{\sum_{n=1}^{N}{\left(u_{p}(n)-{\bar{u}}_{p}\right)}^{2}}\sqrt{\sum_{n=1}^{N}{\left(u_{q}(n)-{\bar{u}}_{q}\right)}^{2}}}$$

where *u_q_*, *u_p_* are the averages of the pluralities ${\bar{u}}_{q}$ and ${\bar{u}}_{P}$. The implementation process of the FMD method is shown in Figure 1. Step 1: Input the original signal *x* and configure the FMD decomposition parameters (*n*, *L*, *K*). Step 2: Initialize the FIR filter bank with *K* filters through the Hanning window, iterating from *i* = 1. Step 3: Obtain the filtered modal components by $u_{k}^{i}$= *x* ∗ $f_{k}^{i}$, where *k* = 1, 2, …, *K*, and ∗ is the convolution operation. Step 4: Update the filter coefficients based on the signal *x*, the decomposed modal component $u_{k}^{i}$, and the fault period $T_{k}^{i}$ (the first local maxima moment after the zero crossing of the autocorrelation spectrum of $u_{k}^{i}$), and the iteration count i is incremented. Step 5: If the iteration count does not reach the upper limit, then return to step 3; otherwise, continue the subsequent process. Step 6: Construct the inter-modal correlation coefficient matrix *CC*, select the modal pair with the largest *CC* to calculate the correlation cliff *CK*, filter the largest *CK* as the valid component, and update *K* = *K* − 1. Step 7: If the current number of modes does not reach the set value *n*, return to step 3; otherwise, stop the iteration and take the obtained modal components as the final decomposition result of FMD.

#### 2.1.2. WOA-Based Parameter Optimization for FMD

The Whale Optimization Algorithm (WOA) simulates a group hunt of humpback whales in the ocean, finding the optimal outcome through a process of searching; encircling, capturing, and attacking prey like a whale population. The WOA provides a mathematical model for encircling, spiraling, and searching prey. The initial position of each whale represents the feasible solution. Through iterative exploration, the algorithm converges to the optimal position, which corresponds to the best solution. In this study, the WOA was employed to optimize the parameters [*n*, *L*, *K*] of the FMD and identify the optimal parameter values. The most important aspect of this method is the selection of a fitness function. In this study, the crest factor of envelope spectrum (*E_c_*) is selected as the adaptability function, *E_c_* is the ratio of the peak value of the envelope signal to the RMS value, and in the mine environment of high noise and multi-condition coupling, by suppressing the local abnormal peaks in the envelope spectrum caused by noise, the periodic shock signal of the fault characteristics is directly retained to avoid the loss of fault characteristics caused by the global smoothing of a traditional entropy value or energy index [27]. Compared with a single peak indicator, the envelope spectrum peak factor can comprehensively assess the abnormal distribution of the overall envelope spectrum of the signal, effectively compressing the dynamic range of noise. *E_c_* value is calculated as shown in Equation (1). Assuming that the signal envelope spectrum amplitude sequence is X(*a*) (*a* =1, 2, ⋯, *A*), we have:

(7) $$E_{C}=\frac{\max\left(X\left(a\right)\right)}{{\left[\frac{\sum_{a}X^{2}\left(A\right)}{A}\right]}^{\frac{1}{2}}}$$

**Figure 2:**
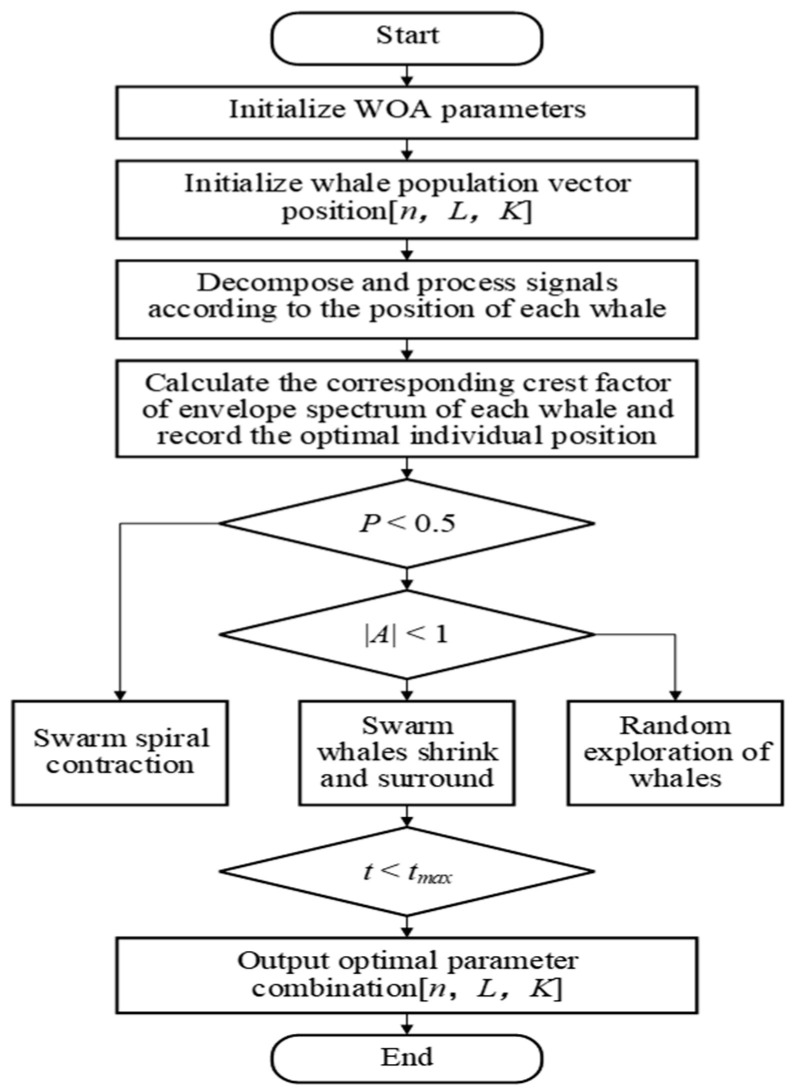
WOA optimization of FMD parameters flowchart.

Figure 2 illustrates the procedure for optimizing the FMD parameters using the WOA. First, the position vector of the whale population [*n*, *L*, *K*] was initialized, followed by the calculation of the fitness value (*E_c_*) for each individual in the population. The positions are then updated based on the iterative formula, and adjustments are applied using the convergence factor throughout the iterative process until the predefined termination condition is satisfied, ultimately obtaining the optimal FMD parameter combination.

#### 2.1.3. Vibration Signal Reconstruction Based on Kurtosis of Squared Envelope Spectrum 

At the time of fault occurrence, the transient energy changes in the signal are mainly affected by the fault and noise impulses. Compared to the traditional kurtosis index, the kurtosis of the square envelope spectrum (KSES) can effectively distinguish the periodic transient components from other components and highlight the fault cyclic impact components in the signal. After decomposing the signal using FMD, the KSES value serves as an indicator: a larger KSES value signifies that the IMF contains richer fault information. The KSES values of each IMF component were calculated, and the average of all these KSES values was adopted as a threshold to select the IMF components for signal reconstruction.

Assuming that an IMF component is labeled *w*, its KSES value can be calculated using the following three steps:

1. Calculate the squared envelope of *w* according to Equation (8), where *i* is an imaginary unit and Hilbert (*w*) denotes the Hilbert transform of *w*.

(8) $$SE\left(w\right)={\left|w+i\cdot Hilbert\left(w\right)\right|}^{2}$$

2. Calculate the squared envelope spectrum of *w* according to Equation (9), where *DFT*[·] denotes the discrete Fourier transform of the *SE*(*w*).

(9) $$SES\left(w\right)=\left|DFT\left[SE\left(w\right)\right]\right|$$

3. Calculate the KSES value of *w* according to Equation (10), where *E*[·] denotes the mathematical expectation to find the long-term average.

(10) $$KSES\left(w\right)=\frac{E\left\{{\left(SES(w)-E\left[SES\left(w\right)\right]\right)}^{4}\right\}}{E{\left\{{\left(SES\left(w\right)-E\left[SES\left(w\right)\right]\right)}^{2}\right\}}^{2}}$$

### 2.2. 2D Time–Frequency Image Generation Based on CWT

For the reconstructed signal, the CWT is employed to generate time–frequency images. This is because CWT can more accurately capture the local variations of the signal in the time–frequency domain compared to grayscale images and envelope spectrograms, thereby offering a more precise representation of the signal features. Let $\psi \left(t\right)\in K^{2}\left(R\right)$, $\psi \left(t\right)$ be a mother wavelet and the wavelet function be stretched and shifted to obtain a continuous wavelet function, as shown in Equation (11).

(11) $$\psi_{a,b}\left(t\right)=\frac{1}{\sqrt{a}}\psi \left(\frac{t-b}{a}\right),a>0,b\in R$$

where *a* is the scaling factor and *b* is the translation factor.

For a square-integrable signal $f\left(t\right)$, when $f\left(t\right)\in K^{2}(R)$, its continuous wavelet transform can be expressed as:

(12) $$C_{f}\left(a,b\right)=\frac{1}{\sqrt{a}}\int f\left(t\right)\psi^{\prime }\left(\frac{t-b}{a}\right)=\int f\left(t\right)\psi_{a,b}^{\prime }\left(t\right)dt=\langle f\left(t\right),\psi_{a,b}\left(t\right)\rangle$$

When *C_f_* (*a*, *b*) is inverted, it follows:

(13) $$f\left(t\right)=\frac{1}{C_{\psi }}\int_{-\infty }^{+\infty }\psi_{a,b}\left(t\right)C_{x}\left(a,b\right)\frac{1}{a^{2}}dadb$$

where:

(14) $$C_{\psi }=2\pi \int_{-\infty }^{+\infty }\frac{\left|\psi \left(\omega \right)\right|}{\left|\omega \right|}d\omega$$

A larger value of parameter *a* is more appropriate for extracting low-frequency features from signals, whereas a smaller *a* tends to be better suited for high-frequency feature extraction. The selection of the basis wavelet function in the wavelet transform significantly influences its performance in terms of effectiveness and efficiency. In this study, the Morlet wavelet was selected as the basis function for the CWT.

### 2.3. Dual-Channel Fault Identification Model Based on RepLKNet-BiGRU-Attention

In this study, we designed a method that integrates a two-dimensional model with a one-dimensional model. By utilizing the RepLKNet module and the BiGRU-Attention module, feature extraction is performed on the time–frequency image signal and one-dimensional time-series signal, respectively, and then feature splicing is performed along the channel dimensions to obtain the fused features, which makes full use of the complementary nature of the two types of data.

**Figure 3:**
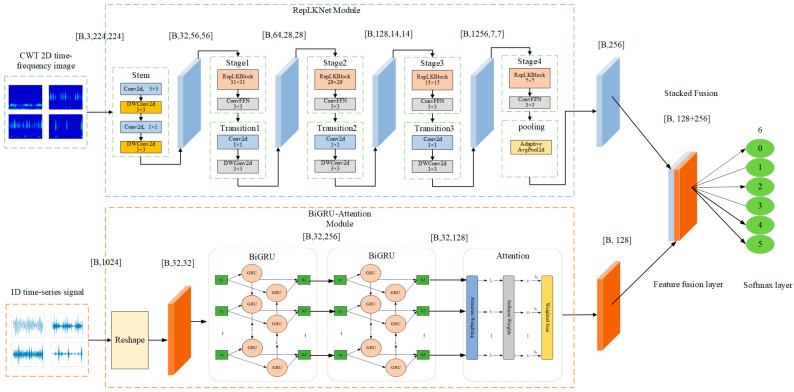
RepLKNet-BiGRU-Attention model structure.

This dual-channel data fusion approach enables the model to simultaneously extract the spatiotemporal features of the data, significantly improving fault classification accuracy. The structure of the model is illustrated in Figure 3. The image branch employs the RepLKNet network (with four-level large convolutional kernels of 31 × 31, 29 × 29, 15 × 15, and 7 × 7) for the progressive feature extraction and downsampling of the input image and ultimately outputs 256-dimensional global feature vectors. The temporal branch reconstructs vibration signals with a length of 1024 into 32 × 32 matrices, and after capturing bi-directional temporal dependencies by a multilayered BiGRU, it generates 128-dimensional features through the attention mechanism to focus the critical fault segments to generate 128-dimensional features. The dual-channel features are stacked and fused into 384-dimensional vectors at the splicing layer, and finally, the fault classification is completed by the fully connected layer.

#### 2.3.1. RepLKNet Module

**Figure 4:**
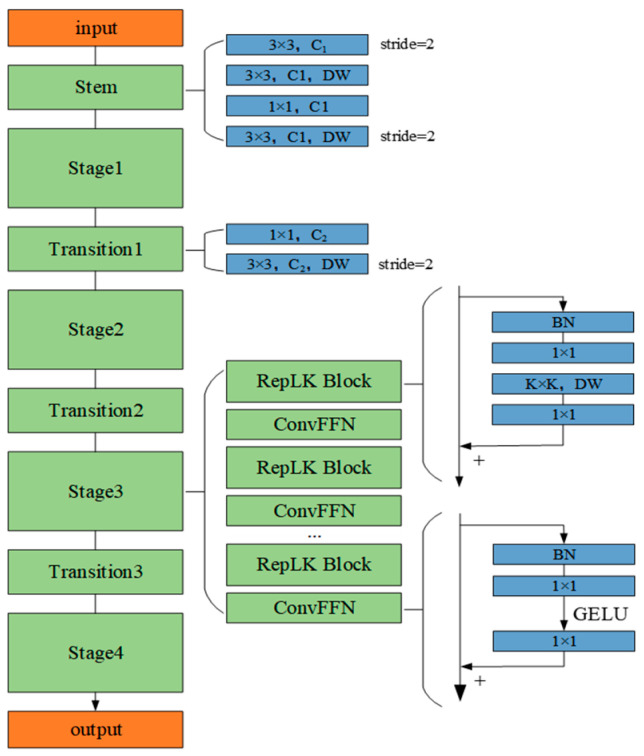
RepLKNet model structure.

To process 2D image signals, this study adopts the RepLKNet model [28] to extract image features. The RepLKNet model introduces large convolution kernels in the RepLKBlock, with sizes of 31 × 31, 29 × 29, 15 × 15, and 7 × 7, respectively. Compared with small kernel convolution, large kernel convolution can cover a larger receptive field in one convolution operation, thus establishing long-range dependencies. In contrast, small kernel convolution usually expands the receptive field gradually by stacking the convolution layers; however, this method may lead to a limited effective receptive field, resulting in feature loss. In the time–frequency feature maps of fault signals, the features are usually continuous and sparsely distributed; therefore, the large kernel convolution has a strong advantage in capturing these critical features. The structure of the model is shown in Figure 4.

The network input layer (stem) adopts a four-stage convolutional layer cascade structure to enhance the detail capturing capability, and the main framework contains four stages (Stage1–Stage4), each of which consists of multiple RepLK blocks bridged by ConvFFN modules. Among them, RepLKBlock adopts a reparameterized 5 × 5 depth separable convolution (DWConv) as the core unit and embeds 1 × 1 convolutional layers before and after it to increase the network depth, strengthen the nonlinear representation, and promote the cross-channel information interaction, while the ConvFFN module fuses batch normalization (BN) and dual 1 × 1 convolutional layers (with the activation function of GELU) to significantly improve the inference efficiency through the BN-convolution integration strategy. The stages are connected by a transition block, which utilizes a 1 × 1 convolution to achieve channel expansion, and a two-stage series-connected 3 × 3 depth-separable convolution to implement progressive downsampling, which reduces computational complexity while maintaining the sensory field and avoiding feature loss.

#### 2.3.2. BiGRU-Attention Module

**Figure 5:**
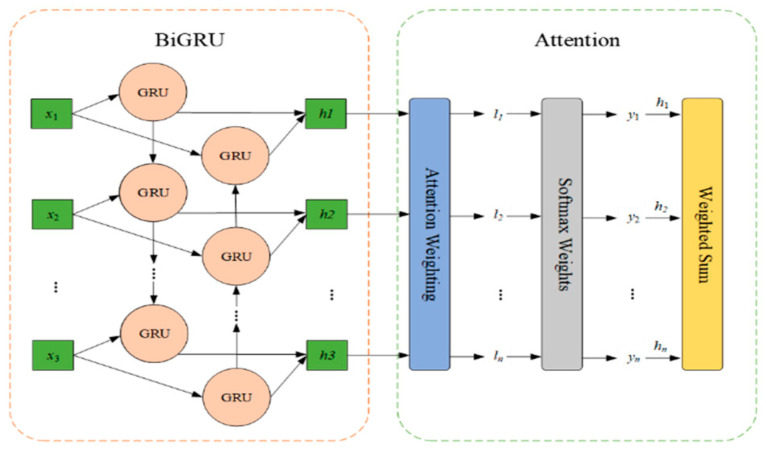
BiGRU-Attention model structure.

When dealing with one-dimensional time-series signals, this study adopts the BiGRU-Attention model to extract time-series features. BiGRU can capture both the forward and backward dependencies of vibration signals through bidirectional processing, which enables the model to better capture the hidden temporal features in time series data. To further enhance the feature extraction, an attention mechanism was also incorporated. By dynamically assigning importance weights to different time steps, the model pays more attention to critical moment information and ignores noisy or unimportant time periods. This design makes BiGRU more effective in extracting key features at the time of fault occurrence when dealing with one-dimensional time-series signals. Figure 5 illustrates the network structure.

BiGRU is constructed by a forward GRU network and a reverse GRU network together. The input sequences are computed in opposite directions in the 2-layer GRU network. Eventually, the outputs of the 2-layer network are integrated according to their respective positions to obtain the final output of the BiGRU network. The attention mechanism is used to assign different weight values to the sequences according to their importance. The corresponding weights are obtained by calculating the similarity between the query and the key, and then these weights are weighted and summed to obtain the final attention value. The output of the BiGRU layer is used as the state matrix and the weight matrix is obtained by random initialization. Normalization is performed using the softmax function to generate the probability distribution, which in turn leads to the final result.

## 3. Fault Diagnosis Framework of This Study

**Figure 6:**
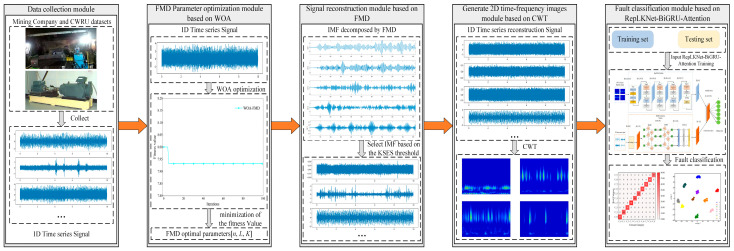
Motor fault diagnosis framework flowchart.

This study proposes a motor fault diagnosis method that integrates WOA-FMD and a RepLKNet-BiGRU-Attention dual-channel network, enabling efficient diagnosis under strong background noise. The specific process is illustrated in Figure 6.

Step 1: Fine-tune the core parameters of the FMD using the WOA to adaptively decompose the fault signal.

Step 2: Analyze the KSES value of each IMF and select the IMF that contains rich fault information using the set threshold for signal reconstruction.

Step 3: Use CWT to convert the reconstructed 1D signal into a 2D image. Then, input both the signal and the image into the RepLKNet-BiGRU-Attention dual-channel network for fault classification.

Step 4: In the model training stage, the results of the training set are used to optimize the weights of the model, and the test set is employed to evaluate the fault detection ability of the model and visualize the results. After several iterations of training and testing, the best-performing network model was selected for efficient fault diagnosis.

## 4. Experimentation and Analysis

The method proposed in this study was applied to the CWRU bearing dataset and real operating data of industrial motors to verify its effectiveness in fault diagnosis under strong background noise. All models in this paper were trained and tested on a PC with a single CPU (AMD Ryzen 7 5800H) and a single GPU (NVIDIA GeForce RTX 3060) running Windows 11, The experimental code was written in Python (Version 3.10) and the deep learning framework was PyTorch (Version 1.13.1) with CUDA (Version 11.7) to utilize GPUs for accelerated computation.

### 4.1. Case 1: Public Dataset

#### 4.1.1. Experimental Data Sources

**Figure 7:**
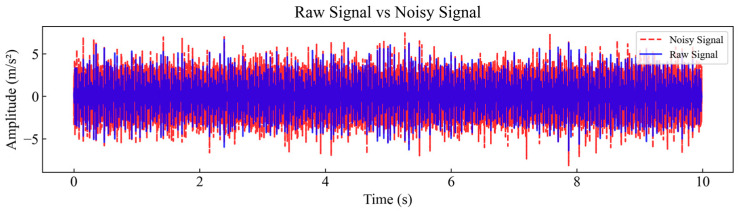
Raw signal and noisy signal in a strong noise environment of −6 dB.

In the course of this experiment, the CWRU dataset furnished by the CWRU Electrical Laboratory was employed. The sampling frequency was 12 kHz, and 119,808 sampling points were selected for each fault type. Healthy data collected at a motor speed of 1797 r/min with a load coefficient of 0 HP, along with nine types of bearing fault data, were included, resulting in a total of 10 types of vibration data. Detailed information about the dataset is shown in Table 1. As the signal test environment is more ideal, the signal contains less noise, and to be closer to the real industrial environment, noise processing is added to all sample data. The added Gaussian white noise ranges from −6 to 6 dB, with varying noise intensity from strong to weak, to evaluate the performance of the proposed method under different levels of background noise. Figure 7 compares the raw and added noise signals of the acceleration vibration signal of the drive end with an outer ring failure diameter of 0.5334 mm in a −6 dB noise environment.

#### 4.1.2. Signal Decomposition and Reconstruction Based on WOA-FMD

**Figure 8:**
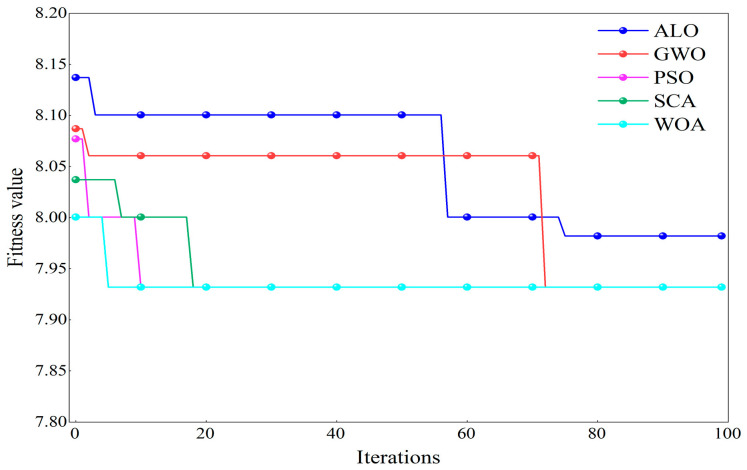
Comparative results of different optimization algorithms.

Taking the signal of an outer race failure with a diameter of 0.5334 mm under a −6 dB strong noise environment as an example, to verify the performance of WOA in optimizing FMD parameters, the optimization results of five different algorithms were compared, and their iteration curves are shown in Figure 8. The WOA algorithm achieved a fitness value of 7.93 after six iterations and then stabilized. In contrast, the other four optimization algorithms exhibited lower optimization speeds and accuracies, with issues such as being prone to local optima and slow convergence.

After WOA optimization, the optimal decomposition parameters were obtained and then applied to the FMD method to decompose the raw signal. The KSES value of each IMF and the mean value of the KSES of all IMF components after decomposition were calculated, and the component with a KSES value larger than the mean value was selected as the dominant IMF to reconstruct the raw signal. The KSES values of each fault type and the dominant IMF of each filtered fault signal are listed in Table 2.

**Figure 9:**
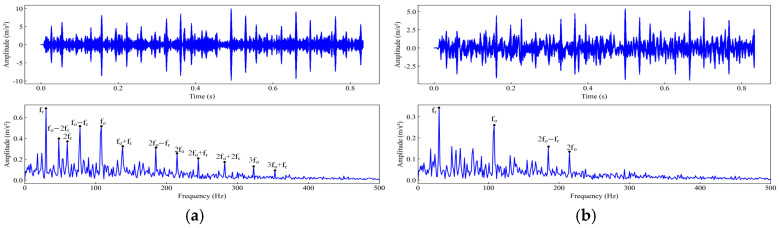

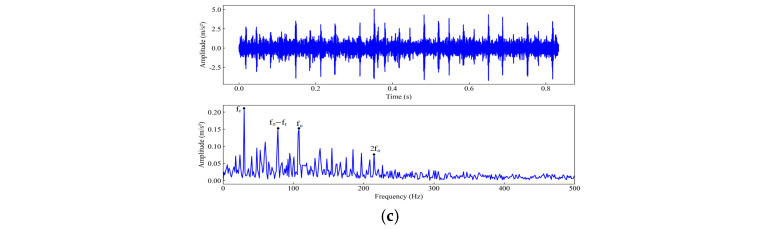
Time–domain spectra and envelope spectra of reconstructed outer ring fault signals using different composition methods: (**a**) WOA-FMD; (**b**) FMD; (**c**) VMD.

To verify the performance of the WOA-FMD method, comparative experiments were carried out using VMD and FMD as benchmark approaches. The decomposition parameters for both VMD and FMD were determined empirically. Taking the outer ring fault signal with a 0.5334 mm fault diameter in a −6 dB strong-noise environment as an example, the bearing outer ring fault characteristic frequency was 107.36 Hz, and the rotational frequency was 29.95 Hz. The IMF components decomposed using the three methods were filtered and reconstructed according to the method described in the text. Figure 9 shows the time–domain spectra and envelope spectra of the signals reconstructed using three methods. Comparative analysis shows that the three methods can all identify the rotational frequency of the signal, as well as fault characteristic frequencies and harmonic components. Nevertheless, the envelope spectrum processed by FMD only displays the fault characteristic frequency and its second harmonic. For the VMD-processed envelope spectrum, only the fault characteristic frequency is discernible, with the second harmonic obscured by spurious frequencies, which results in less distinct fault features. By contrast, the WOA-FMD method effectively suppresses both spurious frequency components and noise in the envelope spectrum, enabling clear distinguishability of the fault frequency and its higher-order harmonics. In summary, when compared with VMD and FMD, the WOA-FMD approach exhibits superior capability in extracting fault features under conditions of strong background noise.

**Figure 10:**
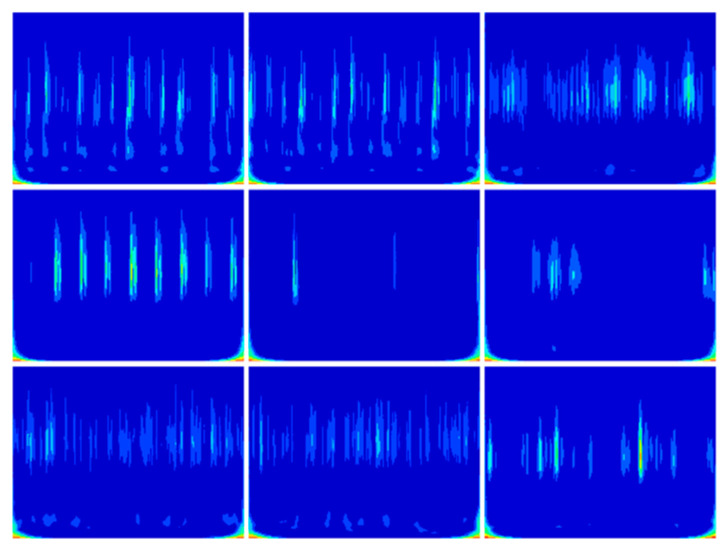
CWT time–frequency image.

The reconstructed signals were re-sampled after min–max normalization according to a length of 1024 and a sliding window overlap rate of 0.5, and 233 training samples were obtained. Then, 233 2D time–frequency images were generated by the CWT. The time–frequency plots generated for several of these samples are shown in Figure 10. Following this method to process all 10 classes of fault samples, a CSV file of 2330 × 1025 was finally generated, with the last column as a label and 2330 2D time–frequency images to form a new dataset.

#### 4.1.3. Dual-Channel Fault Identification Model Based on RepLKNet-BiGRU-Attention

**Figure 11:**
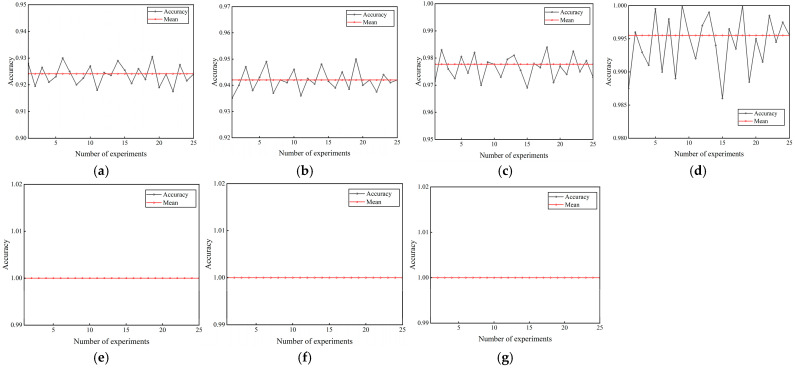
The results of 25 repeatable experiments of the proposed model: (**a**) −6 dB; (**b**) −4 dB; (**c**) −2 dB; (**d**) 0 dB; (**e**) 2 dB; (**f**) 4 db; (**g**) 6 dB.

**Figure 12:**
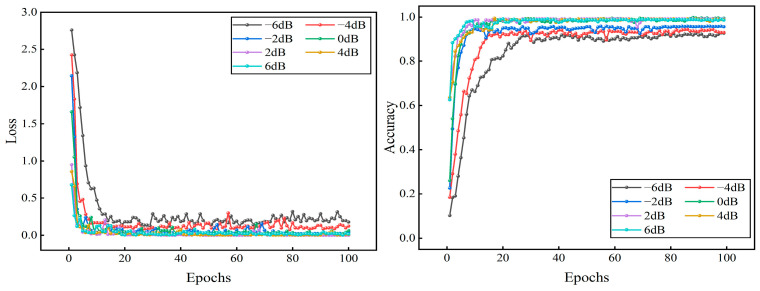
Model loss and accuracy curves on the test set under different noise environments.

**Figure 13:**
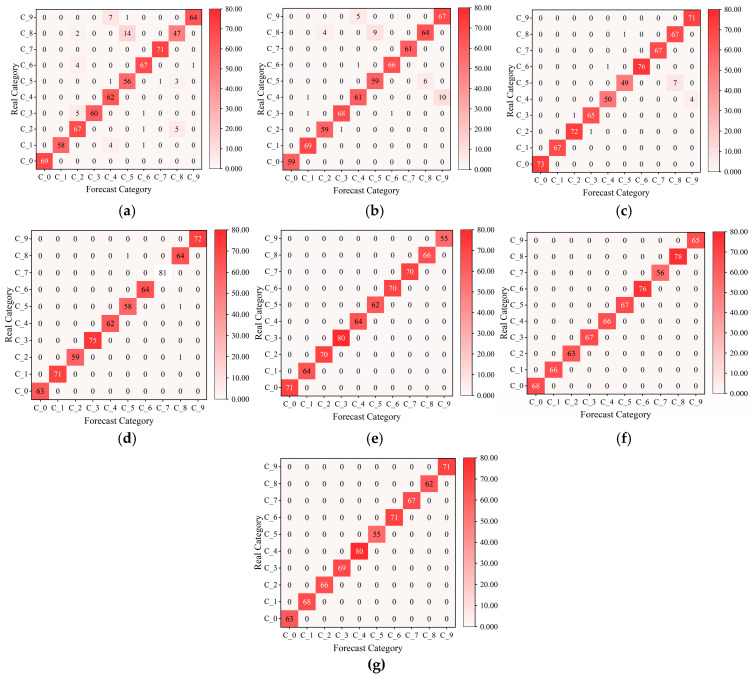
Confusion matrices of the test set under different noise environments: (**a**) −6 dB; (**b**) −4 dB; (**c**) −2 dB; (**d**) 0 dB; (**e**) 2 dB; (**f**) 4 db; (**g**) 6 dB.

**Figure 14:**
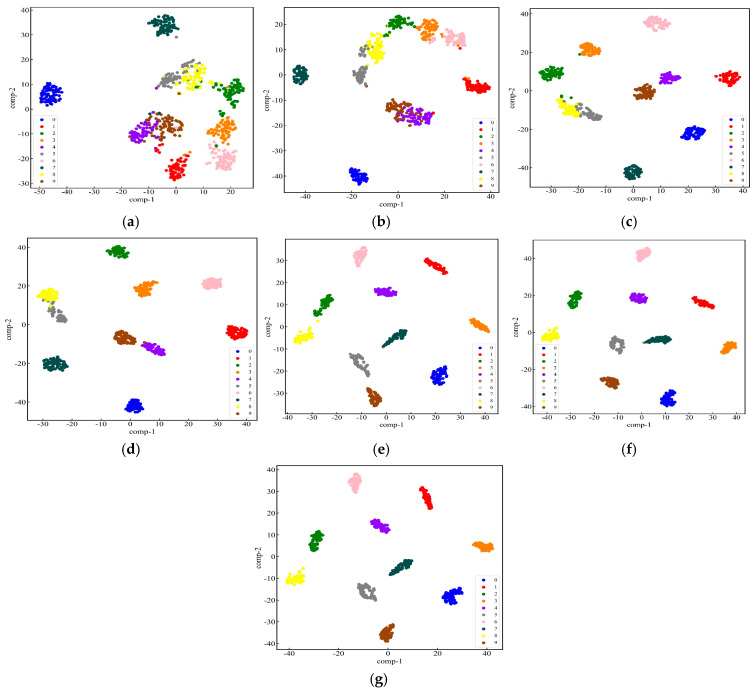
t-SNE dimensionality reduction plots of the test set under different noise environments: (**a**) −6 Db; (**b**) −4 dB; (**c**) −2 dB; (**d**) 0 dB; (**e**) 2 dB; (**f**) 4 db; (**g**) 6 dB.

The newly generated dataset was split into training and test sets in a 7:3 ratio. After multiple experimental comparisons, the parameter optimizer was set to the Adam optimizer, with the cross-entropy loss as the loss function. The learning rate was set to 0.0003, with a batch size of 32, a dropout rate of 0.3, and 100 iterations for the model training. In order to be more in line with the real data flow characteristics of industrial online monitoring scenarios, shuffle = true and drop last = true are set when the test set data is loaded, and the batch will be discarded when the sample size of the last batch of the test set is less than 32, so there will be a case of an unequal number of samples for each label, and since the samples have been randomly disrupted, the proportion of sample discarding for each category will be basically the same as its original distribution, so it will not have a substantial impact on the model evaluation results. Seven datasets in the −6-6 dB noise environment were input into the model for fault identification. The model size is 21.01 MB, the total number of parameters is 5,506,292, the total training time is 803 s, and the average training time per epoch is 8.03 s. In order to verify the stability of the model in this paper, 25 repetitive tests were conducted to validate the model. The final results are shown in Figure 11. It can be seen that the model proposed in this paper maintains high stability under 25 repetitive tests, and the highest diagnostic accuracy and the lowest diagnostic accuracy in each noise environment differ by 1.3%, 1.5%, 1.5%, 1.4%, 0%, 0%, 0%, respectively. A better-performing group is selected and its loss and accuracy curves are plotted. From Figure 12, it can be seen that under −6 dB high-intensity noise, the model still manages to converge completely after 35 epochs with an accuracy of 92.41% ± 0.43%, and as the noise intensity gradually becomes weaker, the model converges faster and faster with an accuracy of 100%. The confusion matrix and t-SNE downscaling distribution plot are used to visualize the fault identification results, as shown in Figure 13 and Figure 14. The differences between different classes of samples can be clearly observed, and the high-frequency confusion mainly occurs between rolling element faults with diameters of 0.3556 mm and 0.5334 mm; the higher the noise, the higher the number of confused samples. Since the eigenfrequency fundamental frequency is the same for both sizes of rolling-body faults, when the noise level increases, the eigenpeaks in the spectrum are swamped by the noise, resulting in the blurring of otherwise distinguishable frequency details. However, in noisy environments above 0 dB, all fault types are perfectly recognized.

**Figure 15:**
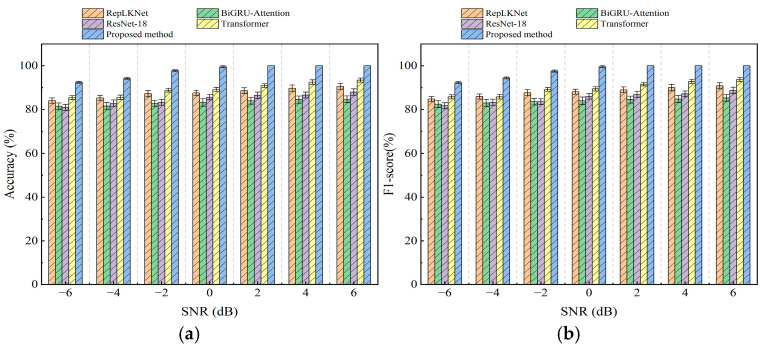
Comparison of model accuracy and F1-score on the test set under different noise environments: (**a**) accuracy; (**b**) F1-score.

To further evaluate the diagnostic capability of the proposed model, its recognition results were compared with those of four other models: RepLKNet, BiGRU-Attention, ResNet-18, and Transformer. The variability of several models is shown in Table 3. The input data of ResNet-18 and RepLKNet are 2D image data, and the input data of Transformer and BiGRU-Attention are 1D data. Accuracy and F1-score were used as evaluation metrics. Twenty-five repetitions of the experiment were performed for all models, and the average accuracy and variance were calculated. The comparison results are shown in Figure 15. The average diagnostic accuracy of this study’s model under different noise intensities was 97.70%, which was 10.06%, 14.54%, 12.86%, and 8.29% higher than that of RepLKNet, BiGRU-Attention, ResNet-18, and Transformer, respectively. The average F1-score was 97.68%, which was 9.64%, 11.76%, 12.36%, and 7.94% higher than that of RepLKNet, BiGRU-Attention, ResNet-18, and Transformer, respectively. The accuracy and F1-score of the RepLKNet-BiGRU-Attention-based two-channel model proposed in this study are the highest under any noise intensity. This proves that the proposed model achieves higher fault identification accuracy and greater robustness under different noise environments.

#### 4.1.4. Analysis of Experimental Results

**Figure 16:**
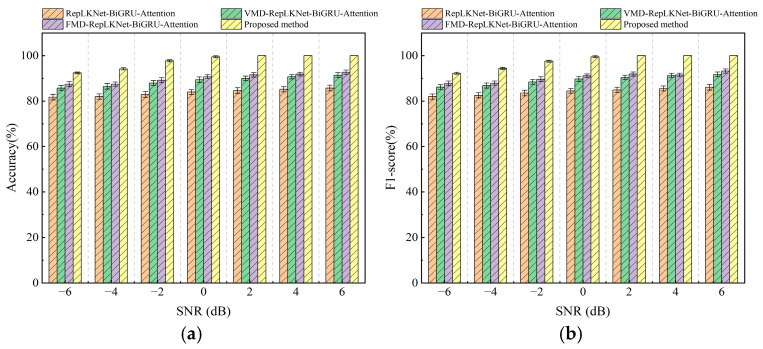
Comparison of method accuracy and F1-score on the test set under different noise environments: (**a**) accuracy; (**b**) F1-score.

To verify the comprehensive performance of this method, the method was compared with the other three methods by using the original signals with added noise. The variability of several methods is shown in Table 4. As illustrated in Figure 16, the average diagnostic accuracy of the proposed method is 97.70%, which is 13.95%, 8.88%, and 7.56% higher than that of RepLKNet-BiGRU-Attention, VMD-RepLKNet-BiGRU-Attention, and FMD-RepLKNet-BiGRU-Attention, respectively. The average F1 score is 97.68%, which is 13.53%, 8.54%, and 7.23% higher than that of RepLKNet-BiGRU-Attention, VMD-RepLKNet-BiGRU-Attention, and FMD-RepLKNet-BiGRU-Attention, respectively. Under any noise intensity, the accuracy and F1 score of the dual-channel model based on RepLKNet-BiGRU-Attention are the highest. This demonstrates that the proposed method has high applicability and superiority in different noise environments.

### 4.2. Case 2: Actual Operational Data

#### 4.2.1. Experimental Data Sources

**Figure 17:**
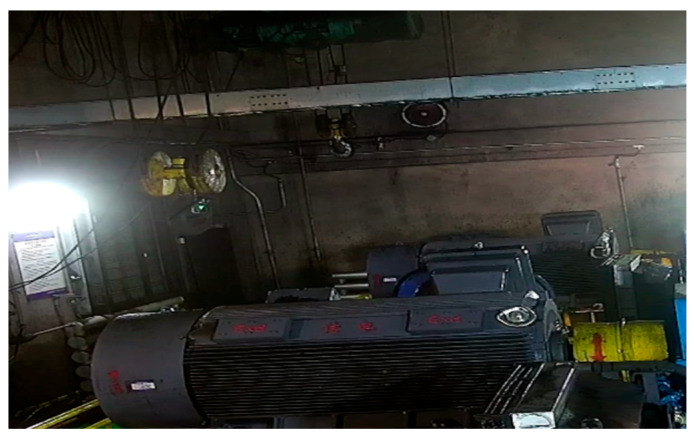
Motor for the mine conveyor belt.

The experimental data are from the driving system of the belt conveyor on Line 2 of the surface production system in a large-scale open-pit coal mine. This belt conveyor is powered by three YB3-450-4W three-phase asynchronous motors, as shown in Figure 17. The motor speed was 1480 r/min, the vibration data sampling frequency was 5 kHz, and 163,840 sampling points were selected for each fault type. The data from when the motor is under normal operation are selected as the health data, as well as the bearing retainer, bearing outer ring, bearing rolling element, shaft misalignment, shaft imbalance, and five types of fault data, totaling six types of data. Table 5 shows the types of faults and their corresponding labels.

#### 4.2.2. Signal Decomposition and Reconstruction Based on WOA-FMD

**Figure 18:**
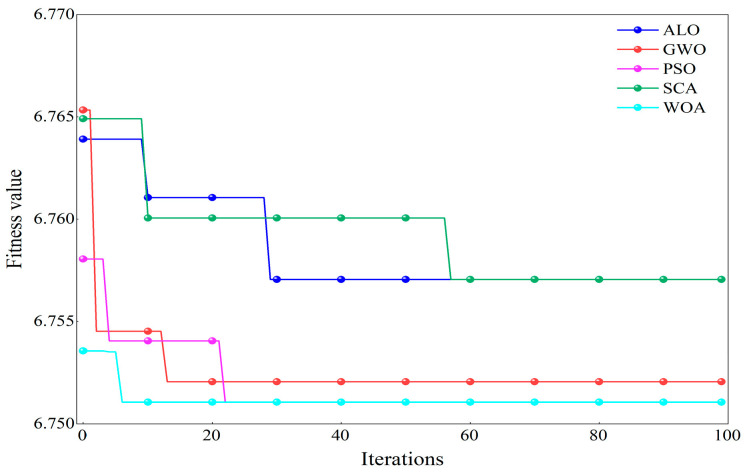
Comparative results of different optimization algorithms.

Taking the outer ring fault of the bearing of a mine-used motor as an example, to verify the performance of WOA in optimizing FMD parameters, the optimization results of five different algorithms were compared, and their iteration curves are shown in Figure 18. The WOA algorithm achieved a fitness value of 6.75 after eight iterations and then stabilized. In contrast, the other four optimization algorithms exhibited lower optimization speeds and accuracies, with issues such as being prone to local optima and slow convergence.

After WOA optimization, the optimal decomposition parameters were obtained and then applied to the FMD method to decompose the raw signal. The KSES value of each IMF and the mean value of the KSES of all IMF components after decomposition were calculated, and the component with a KSES value larger than the mean value was selected as the dominant IMF to reconstruct the raw signal. The KSES values of each fault type and the dominant IMF of each filtered fault signal are listed in Table 6.

**Figure 19:**
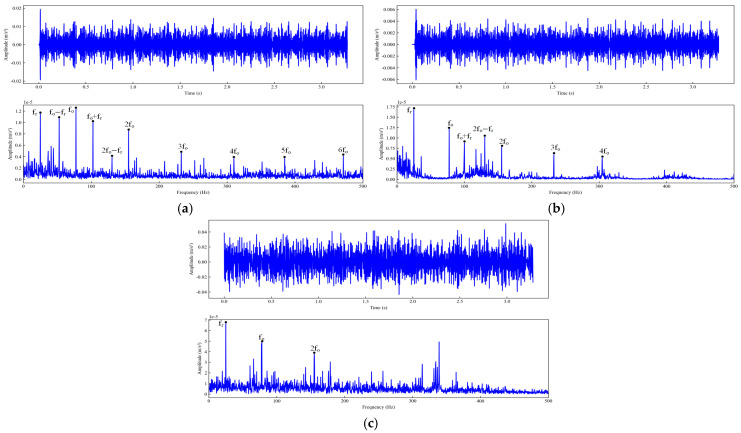
Time–domain spectra and envelope spectra of reconstructed outer ring fault signals using different composition methods: (**a**) WOA-FMD; **(b**) FMD; (**c**) VMD.

To verify the performance of the WOA-FMD method, comparative experiments were carried out using VMD and FMD as benchmark approaches. The decomposition parameters for both VMD and FMD were determined empirically. The bearing outer ring fault characteristic frequency was 77.24 Hz, and the rotational frequency was 24.67 Hz. The IMF components decomposed by the three methods were filtered and reconstructed following the procedure described in this study. Figure 19 shows the time–domain spectra and envelope spectra of the signals reconstructed using three methods. Comparative analysis shows that the three methods can all identify the rotational frequency of the signal, as well as fault characteristic frequencies and harmonic components. However, the FMD-processed envelope spectrum only reveals the fault characteristic frequency and its second to fourth harmonics, while the VMD-processed spectrum shows only the fault characteristic frequency and its second harmonic. Both are accompanied by significant spurious frequency interferences, resulting in indistinct fault features. By contrast, the WOA-FMD method effectively suppresses spurious frequencies and noise in the envelope spectrum, enabling clear visualization of the fault frequency and its second to sixth harmonics. In summary, compared with VMD and FMD, WOA-FMD demonstrates a superior fault feature extraction capability under strong background noise in mining environments.

**Figure 20:**
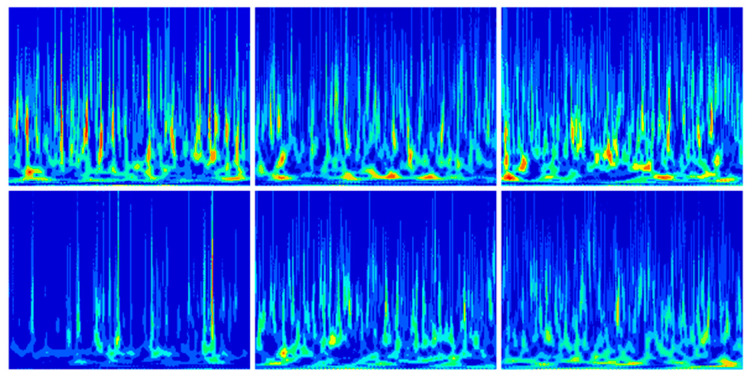
CWT time–frequency image.

The reconstructed signals were re-sampled after min–max normalization according to a length of 1024 and a sliding window overlap of 0.5 to obtain 305 training samples. Then, 305 2D time–frequency images were generated by CWT. The time–frequency plots generated for several of these samples are shown in Figure 20. Following this method to process all six types of fault samples, a CSV file of 1830 × 1025 is finally generated, with the last column as the label and 1830 2D time–frequency images to form a new dataset.

#### 4.2.3. Dual-Channel Fault Identification Model Based on RepLKNet-BiGRU-Attention

**Figure 21:**
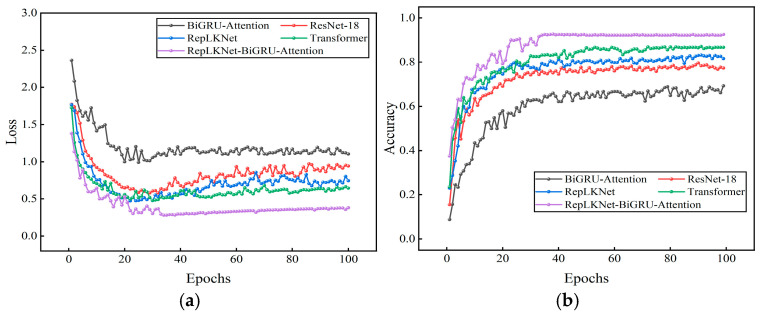
(**a**)Accuracy curve of different models on the test set. (**b**) Loss curve of different models on the test set.

**Figure 22:**
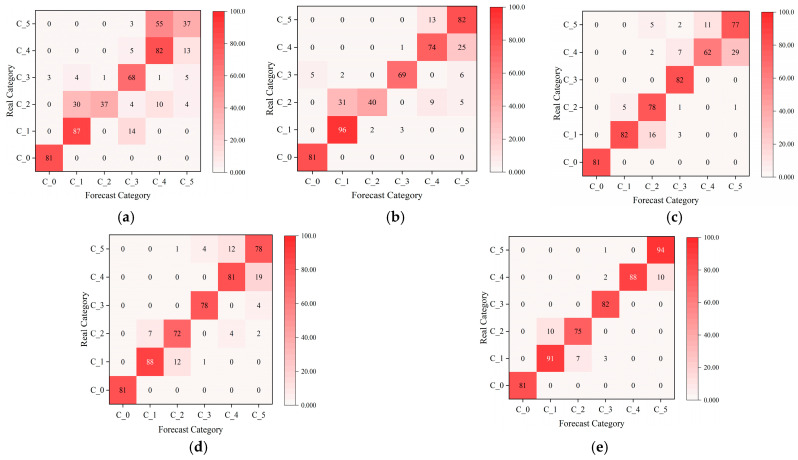
Confusion matrices for different models on the test set: (**a**) BiGRU-Attention; (**b**) ResNet-18; (**c**) RepLKNet; (**d**) Transformer; (**e**) RepLKNet- BiGRU-Attention.

**Figure 23:**
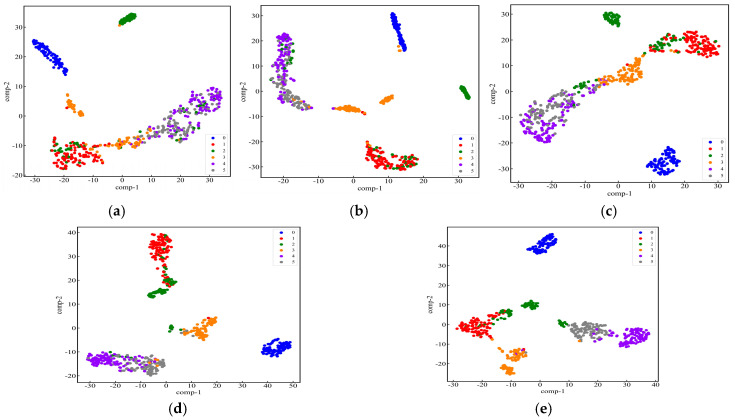
t-SNE dimensionality reduction plots of different models on the test set: (**a**) BiGRU-Attention; (**b**) ResNet-18; (**c**) RepLKNet; (**d**) Transformer; (**e**) RepLKNet- BiGRU-Attention.

The dataset and model parameters are divided in the same proportion as in Case 1. The model recognition results were compared with four other models: RepLKNet, BiGRU-Attention, ResNet-18, and Transformer. The input data of ResNet-18 and RepLKNet are 2D image data, and the input data of Transformer and BiGRU-Attention are 1D data. Twenty-five replicate experiments were performed on all models, and mean accuracy and variance were calculated. A set of experiments with better results for each model was selected to plot the iterative curves of loss and accuracy, as shown in Figure 21. It can be seen that with the increase in the number of iterations, the loss value and accuracy of each model gradually converge and tend to be stable. Table 7 shows the accuracy and F1 score for each model. Compared with several other models, the model in this study had the highest average accuracy and average F1-score, and the average accuracy rate is higher than BiGRU-Attention, ResNet-18, RepLKNet, and Transformer by 21.87%, 12.68%, 9.00%, and 6.06%, respectively. The F1-scores are higher than those of 22.54%, 13.37%, 9.25%, and 5.98%. The confusion matrix and t-SNE dimensionality reduction distribution plot are used to visualize and analyze the fault identification results of each model, as shown in Figure 22 and Figure 23. It can be seen that all models are able to recognize label 0 (no fault) perfectly, and ResNet-18 has the highest recognition accuracy for label 1 (bearing rolling element fault). RepLKNet and this paper’s model both achieve 100% recognition accuracy for label 3 (bearing outer ring fault), but this paper’s model has the highest recognition accuracy for label 5 (shaft imbalance fault). Further analyzing the recognition results of all models, it is found that the high-frequency confusion is mainly between label 1 (bearing rolling element fault) and label 2 (bearing retainer fault) and between label 4 (shaft misalignment fault) and label 5 (shaft imbalance fault). This is because, in the complex working environment of mines, dust and noise from coupled equipment operation can lead to an increase in the similarity of signal features, thus interfering with the model identification results. However, the model proposed in this paper has the least number of misclassified samples and the highest classification accuracy. It can be proven that the model proposed in this study can be more accurate and robust for fault identification under the real operating conditions of the mining motor. This also proves that the dual-channel feature extraction is complementary, and the fault information is more complete compared with the single channel, which can extract fault features from multiple scales.

#### 4.2.4. Analysis of Experimental Results

To verify the comprehensive performance of this method, the method was compared with the other three methods by using the raw signals, and the results are shown in Table 8. The average diagnostic accuracy of the proposed method was 93.93%, which was 19.30%, 11.58%, and 7.35% higher than that of RepLKNet-BiGRU-Attention, VMD-RepLKNet-BiGRU-Attention, and FMD-RepLKNet-BiGRU-Attention, respectively, and the F1-score was 93.90%, which was 20.05%, 11.47%, and 7.26% higher than that of RepLKNet-BiGRU-Attention, VMD-RepLKNet-BiGRU-Attention, and FMD-RepLKNet-BiGRU-Attention, respectively. Under the real operating conditions of the equipment, the accuracy and F1-score of the method based on the combination of WOA-FMD feature extraction and the RepLKNet-BiGRU-Attention fault identification model are the highest. This proves the superiority of the fault diagnosis method used in this study in terms of fault feature extraction ability and fault type recognition ability under the real operating conditions of the mining motor.

## 5. Conclusions

Aiming at problems such as the poor ability to extract signal fault features and low accuracy in identifying fault types existing in the fault diagnosis of mine-used motors under strong background noise, a new method of fault diagnosis combining WOA-FMD and RepLKNet-BiGRU-Attention is introduced.

The main research results are summarized as follows:

1. The core parameters of the FMD were adaptively optimized using the WOA, and the KSES value was used as an index to select the dominant IMF components for signal reconstruction. This method can successfully remove irrelevant noise components in the signal and make the fault features more obvious. The core parameters of the FMD were adaptively optimized using the WOA, with the KSES value serving as an index to select the dominant IMF components for signal reconstruction. This method effectively removes irrelevant noise components from the signal, thereby enhancing the visibility of fault features.
2. Fault identification was performed using the RepLKNet-BiGRU-Attention dual-channel deep learning network model. The CWT image preserves the spatial features of the signal, while the one-dimensional signal retains time-dependent features. The RepLKNet-BiGRU-Attention dual-channel model simultaneously extracts multiscale features of the signal for fusion, thereby enhancing the fault recognition accuracy.
3. By combining WOA-FMD with RepLKNet-BiGRU-Attention, a high-performance fault diagnosis method for mining motors was developed.
4. In this study, an experimental analysis was carried out using a noise-processed CWRU bearing dataset and various types of fault data under the actual operation of enterprise mining motors. The results show that, compared with the VMD and FMD methods without parameter optimization, the feature extraction method proposed in this paper can effectively extract the fault characteristic frequencies and their corresponding higher harmonics. Moreover, the introduced fault recognition method demonstrated higher accuracy than the other four methods. These results indicate that the proposed method has significant advantages in the fault diagnosis of mining motors under strong background noise.

In addition, this study discusses parts of the proposed method that can be improved. In the proposed screening of dominant IMFs using KSES indicators, a single screening indicator may be easily disturbed by strong background noise, leading to the problem of election errors. Other indicators can be introduced for secondary screening, or composite screening indicators can be used to make the screening results more accurate.

## Figures and Tables

**Table 1:** Fault data information and classification labels.

Fault Diameter (mm)	Fault Type	Labels
No fault	No fault	0
0.1778	Inner ring fault	1
0.1778	Rolling element fault	2
0.1778	Outer ring fault	3
0.3556	Inner ring fault	4
0.3556	Rolling element fault	5
0.3556	Outer ring fault	6
0.5334	Inner ring fault	7
0.5334	Rolling element fault	8
0.5334	Outer ring fault	9

**Table 2:** KSES values and dominant modal components for each fault type.

Number	Decomposition Number	KSES Value	Mean Value	Dominant IMF
0	10	9.6, 14.1, 11.1, 4.6, 12.3, 8.0, 6.6, 6.1, 8.0, 5.7	8.6	1, 2, 3, 5
1	9	13.6, 7.0, 9.2, 5.2, 11.8, 9.3, 5.8, 7.5, 6.9	8.5	1, 3, 5, 6
2	6	9.7, 8.5, 8.7, 11.4, 16.0, 6.9	10.2	4, 5
3	7	5.8, 9.6, 11.7, 15.1, 7.9, 7.4, 6.4	9.1	2, 3, 4
4	5	9.9, 9.3, 10.4, 9.3, 11.0	10.0	3, 5
5	8	6.6, 9.7, 12.5, 10.0, 13.1, 11.1, 8.6, 9.7	10.2	3, 5, 6
6	7	11.9, 9.8, 10.1, 15.5, 8.7, 6.8, 8.9	10.2	1, 4
7	4	10.2, 6.2, 5.6, 11.9	8.5	1, 4
8	8	6.2, 8.6, 7.2, 10.3, 13.5, 7.4, 7.6, 6.8	8.5	2, 4, 5
9	6	8.3, 6.7, 38.4, 7.8, 7.2, 10.8	13.2	3

**Table 3:** Comparison of the differences between several models.

Models	Input Data Type	Core Structure	Feature Extraction Capability
BiGRU-Attention	1D time-series signal	BiGRU + Attention mechanism	Captures only timing dependencies, lacks spatial feature modeling.
ResNet-18	2D time–frequency image	Residual convolutional network	Strong local spatial features, but easy to ignore temporal dynamic information.
RepLKNet	2D time–frequency image	Large convolution kernel	Large sensory fields capture long-range spatial features but not temporal features.
Transformer	1D time-series signal	Self-attention mechanism	Global timing dependence modeling, but with high computational overhead and insensitivity to local shock characteristics.
Proposed method	1D signal + 2D image	Fusion of spatial and temporal features	Extracting large-scale spatial features while capturing bidirectional temporal dependencies.

**Table 4:** Comparison of the differences between several methods.

Method	Data Preprocessing	Parameter Optimization Mechanism
RepLKNet-BiGRU-Attention	None	None
VMD-RepLKNet-BiGRU-Attention	VMD Decomposition + KSES Reconstruction	Fixed parameters (empirically dependent)
FMD-RepLKNet-BiGRU-Attention	FMD Decomposition + KSES Reconstruction	Fixed parameters (empirically dependent)
Proposed method	WOA-FMD Decomposition + KSES Reconstruction	WOA Adaptive Optimization Dynamic Search for Optimal [*n*, *L*, *K*]

**Table 5:** Motor fault data classification.

Fault Type	Labels
No fault	0
Bearing rolling element fault	1
Bearing retainer fault	2
Bearing outer ring fault	3
Shaft misalignment fault	4
Shaft imbalance fault	5

**Table 6:** KSES values and dominant modal components for each fault type.

Number	Decomposition Number	KSES Value	Mean Value	Dominant IMF
0	6	5.5, 11.0, 17.3, 5.3, 14.2, 6.5	10.0	2, 3, 5
1	10	10.8, 6.4, 9.3, 7.9, 2.9, 6.6, 6.4, 14.9, 3.9, 11.5	8.1	1, 3, 8, 10
2	3	19.3, 9.7, 7.4	12.1	1
3	6	4.4, 8.9, 7.8, 6.1, 6.6, 18.0	8.6	6
4	7	5.2, 7.7, 4.8, 6.5, 7.7, 7.4, 18.2	8.2	7
5	8	5.6, 10.9, 10.1, 17.1, 6.5, 17.4, 10.0, 7.8	10.7	2, 4, 6

**Table 7:** Accuracy and F1-score of different models on the test set.

Models	Accuracy (Mean ± Std)	F1-Score (Mean ± Std)
BiGRU-Attention	0.7206 ± 0.022	0.7136 ± 0.019
ResNet-18	0.8125 ± 0.017	0.8053 ± 0.018
RepLKNet	0.8493 ± 0.014	0.8465 ± 0.012
Transformer	0.8787 ± 0.011	0.8792 ± 0.010
Proposed method	0.9393 ± 0.007	0.9390 ± 0.005

**Table 8:** Accuracy and F1-score of different methods on the test set.

Method	Accuracy (Mean ± Std)	F1-Score (Mean ± Std)
RepLKNet-BiGRU-Attention	0.7463 ± 0.021	0.7385 ± 0.019
VMD-RepLKNet-BiGRU-Attention	0.8235 ± 0.015	0.8243 ± 0.017
FMD-RepLKNet-BiGRU- Attention	0.8658 ± 0.012	0.8664 ± 0.014
Proposed method	0.9393 ± 0.007	0.9390 ± 0.005

## Data Availability

The data presented in this study are available on request from the corresponding author. The data are not publicly available due to confidentiality restrictions (the dataset contains proprietary information from a collaborating enterprise).
